# Clinical and translational science award hub portfolio analysis and interorganizational collaborations with Los Angeles County to improve population health and health care delivery (ID 1565096)

**DOI:** 10.3389/fpubh.2025.1565096

**Published:** 2025-06-19

**Authors:** Pamela L. Davidson, Terry T. Nakazono, Andrea Min, Jim Morrison, Omira Quail

**Affiliations:** ^1^CTSI-Evaluation, Clinical and Translational Science Institute (CTSI), University of California, Los Angeles, Los Angeles, CA, United States; ^2^Department of Health Policy and Management, Fielding School of Public Health, University of California, Los Angeles, Los Angeles, CA, United States; ^3^Bachelor of Science Program, Biology with Minors in Epidemiology and Biostatistics, University of Western Ontario, London, ON, Canada

**Keywords:** translational science, CTSA program, knowledge translation, interorganizational collaborations, hub-county collaborations, TSBM, translational science impact

## Abstract

Demonstrating impact in the Clinical and Translational Science Award (CTSA) Program is crucial to continue governmental, taxpayer, institutional, and donor support and investment. We present an innovative portfolio analysis to summarize the Scientific Achievement Translational Science Impact at the hub level. Additionally, a unique feature of the UCLA CTSA hub includes the many interorganizational collaborations with Los Angeles County (LAC). This is the first study to examine the Translational Science Benefits Model (TSBM) impact on projects with CTSA hub-county interorganizational collaborations. A Framework for Evaluating Scientific Achievement Translational Science Impact (SATSI) was used to guide the analyses, with impact indicators derived from the TSBM: (i) clinical and medical, (ii) community and public health, (iii) economic, and (iv) policy and legislation. Two major data sources were used for the evaluation: (i) The CTSI’s Longitudinal Scientific Achievement and Impact survey (LSAS-I), and (ii) longitudinal interviews with principal investigators who reported high-impact projects in hub-county collaborations. We reported baseline data from 2 years of LSAS-I data showing *n* = 507 new CTSA-assisted grants and the associated demonstrated and potential impact using the hub portfolio analysis. Eighteen (*n* = 18) of these grants involved a hub-county interorganizational collaboration. Among these, we identified the highest impact projects and developed impact stories and vignettes describing improvements in health care delivery and population health. Our research offers a model for other CTSA hubs to summarize impact using the hub portfolio analysis, and to partner with local public health departments and governmental agencies to address health concerns in low-income and at-risk populations. This research directly addresses the mission of the UCLA hub, “to produce and implement innovations that impact the greatest health needs of Los Angeles and the nation.”

## 1 Introduction

The NIH National Center for Advancing Translational Science (NCATS) funds the Clinical and Translational Science Award (CTSA) Program, supporting over 60 hubs across the nation. This study examines the overall knowledge translation (as an intermediate impact measure) of the University of California Los Angeles (UCLA) Clinical and Translational Science Institute (CTSI) using the TSBM: Translational Science Benefits Model (1). In the 2021 CTSA Evaluators Survey, 68% of hubs reported using the TSBM for evaluation impact measurement; by 2024 the percentages had increased to 75.5% of hubs responding to the survey (Hunt, 2025, unpublished)^1^. This Frontiers in Public Health Research Content aims to assess the state-of-the-science in TSBM research and development in the CTSA Program. The TSBM is used to collect demonstrated and potential impact in four domains: (i) clinical and medical, (ii) community and public health, (iii) economic, and (iv) policy and legislative benefits. Demonstrating impact is crucial to expand and sustain stakeholder investment. This paper presents a novel approach for reporting CTSA hub profile analysis to graphically illustrate to funding agencies and institutional donors the Scientific Achievement Translational Science Impact (SATSI).

**Figure 1:**
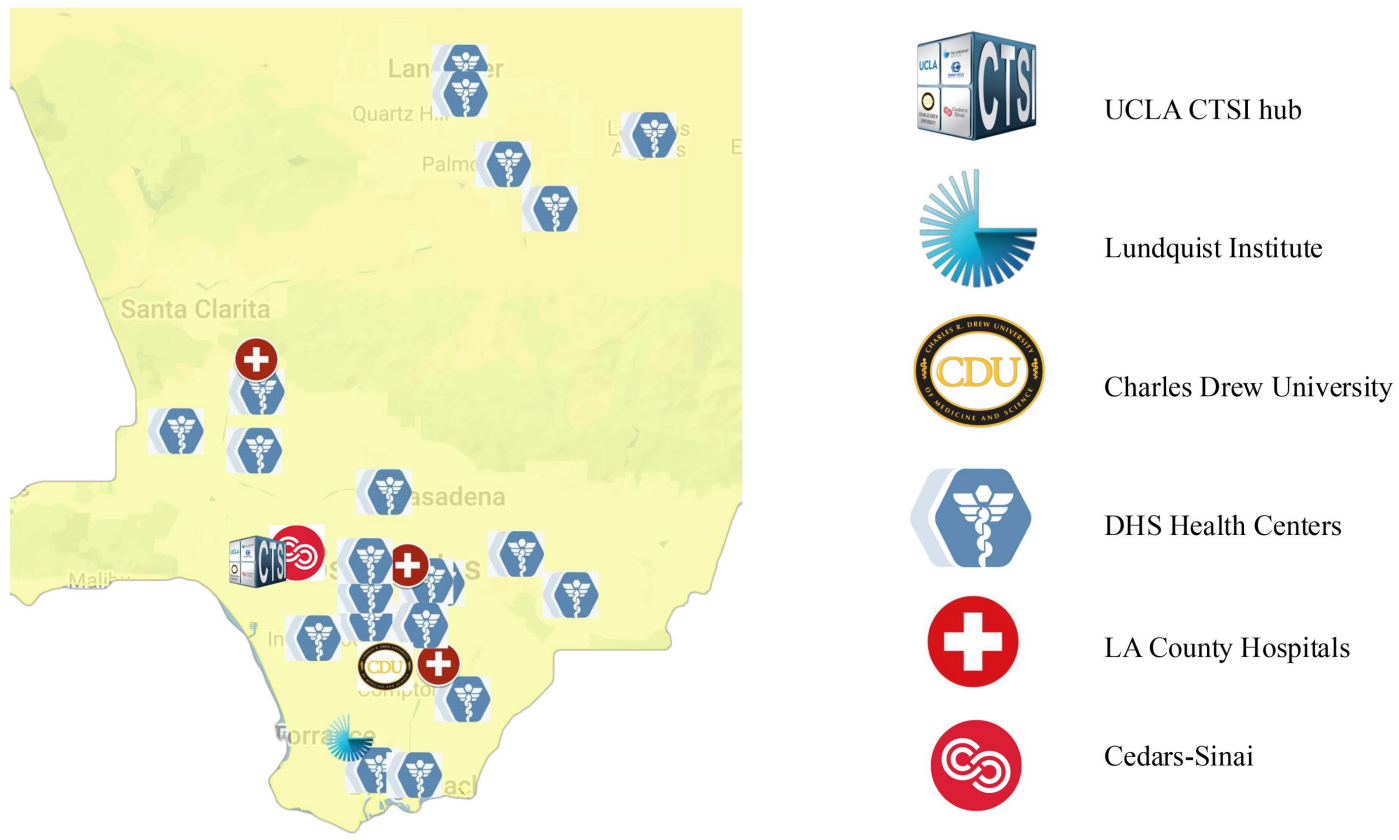
CTSI partner institutions and Los Angeles County health centers and hospitals.

Additionally, this innovative study examines TSBM impact in hub-county interorganizational collaborations; no other studies were found in the peer-reviewed literature that looked at impact in a systematic way using quantitative analysis. Spanning four Los Angeles-based partner institutions (Cedars-Sinai, Charles R. Drew University, Harbor-UCLA/The Lundquist Institute for Biomedical Innovation, and UCLA), the CTSI is involved in interorganizational collaborations with Los Angeles County (LAC) health departments, as well as other county governmental agencies. Figure 1 shows the hub institutional partners along with a sample of the vast opportunities for interorganizational collaborations with LAC. Our research offers a model for other CTSA hubs to partner with local health departments to improve health and healthcare.

## 2 Materials and methods

Methods for this study include: (1) assessing the national context of CTSA hub-county interorganizational collaborations; and (2) CTSI evaluation framework to guide quantitative and qualitative data collection and analysis to examine TSBM knowledge translation impact both overall in the hub portfolio and in hub-LAC interorganizational collaborations.

### 2.1 National context of hub-county interorganizational collaborations

Limited research exists on the extent to which CTSA hubs collaborate with their local city, county, and/or state governmental organizations and no studies were found that examined quantitative data on the impact of these collaborations. Among the available studies which were descriptive, one study concluded that strong public health partnerships improve research dissemination, policy development, and community health outcomes (2), while other studies emphasized the importance of collaboration for advancing public health initiatives, application to rural health initiatives, and the importance of trust in collaborator relationships (3–5). None of the studies collected or analyzed quantitative data on impact.

The justification for conducting this internet research was to provide a context for the hub-county collaborative activity at our CTSA and others across the nation. Since no systematic data were available on the extent of interorganizational collaborations among the CTSA hubs and county health departments, we wanted to understand how our hub compares with other hubs across the nation. We conducted internet research using publicly available website information to count the number of collaborations with county agencies associated with each of the hubs. Content analysis was used to rank CTSA hubs on the level of collaborations with county governmental institutions using a methodology created by Tafuto et al. (2).

To quantify the extent of CTSA hub interorganizational collaborations, we applied a ranking-based content analysis methodology (2), which was originally designed to evaluate and compare CTSA hub websites on the content alignment with NCATS goals and initiatives. The findings from this study were determined using a structured ranking system like Tafuto et al. (2). This system quantifies website content and assigns numerical scores based on defined criteria. In Tafuto’s research, each CTSA hub was evaluated for the presence of relevant content, the variety of content formats, and the navigational ease of accessing this information. These three metrics were merged into a composite score for each hub, allowing for standardized comparisons across all 58 hubs.

In our study, we adapted this ranking framework to systematically categorize and score CTSA hubs on their level of collaboration with county and governmental organizations, using publicly available web data. This approach was essential for the study given the absence of centralized data sources on hub-county partnerships. This approach provided a consistent and replicable method for gauging engagement trends among hubs. The validity of this ranking system has been demonstrated through its prior application to all 58 CTSA hubs in Tafuto’s study, and its structured scoring ensured objective differentiation between varying levels of interorganizational engagement based on information posted on CTSA hub websites.

For our analysis, we constructed an ordinal 4-point scale—ranging from no evidence of collaboration (0) to three or more collaborations (3+)—inspired by Tafuto’s scoring thresholds for content representation showing the intensity of hub-county collaborations. Specifically coded as: (0) No internet evidence suggesting hub-county collaborations, (1) hub had limited (only one) collaboration with county organizations, (2) hub had a moderate level (two) collaborations, and (3) CTSA website showed three or more hub-county collaborations.

Internet publicly available website data search was the most appropriate method since no other data source on hub-county collaborations was found. Sixty-one (*n* = 61) hubs were identified on the NIH CTSA hub directory (6). Each CTSA hub website and/or related content were examined through queries and evaluated for content related to hub-county interorganizational collaborations. Hubs were categorized using a ranking system showing the presence and accessibility of information related to collaborations with county organizations. This allowed us to compare our hub’s activities relative to national peers as well as draw data-driven conclusions about the frequency and intensity of hub-county collaborations across 61 hubs. Hence, this analysis, building on the Tafuto et al. (2) methodology, provides an approach to constructing contextual variables to assess and compare national context, in the absence of a systematic data source (7–10).

### 2.2 Evaluation framework to guide quantitative and qualitative analysis of TSBM impact

**Figure 2:**
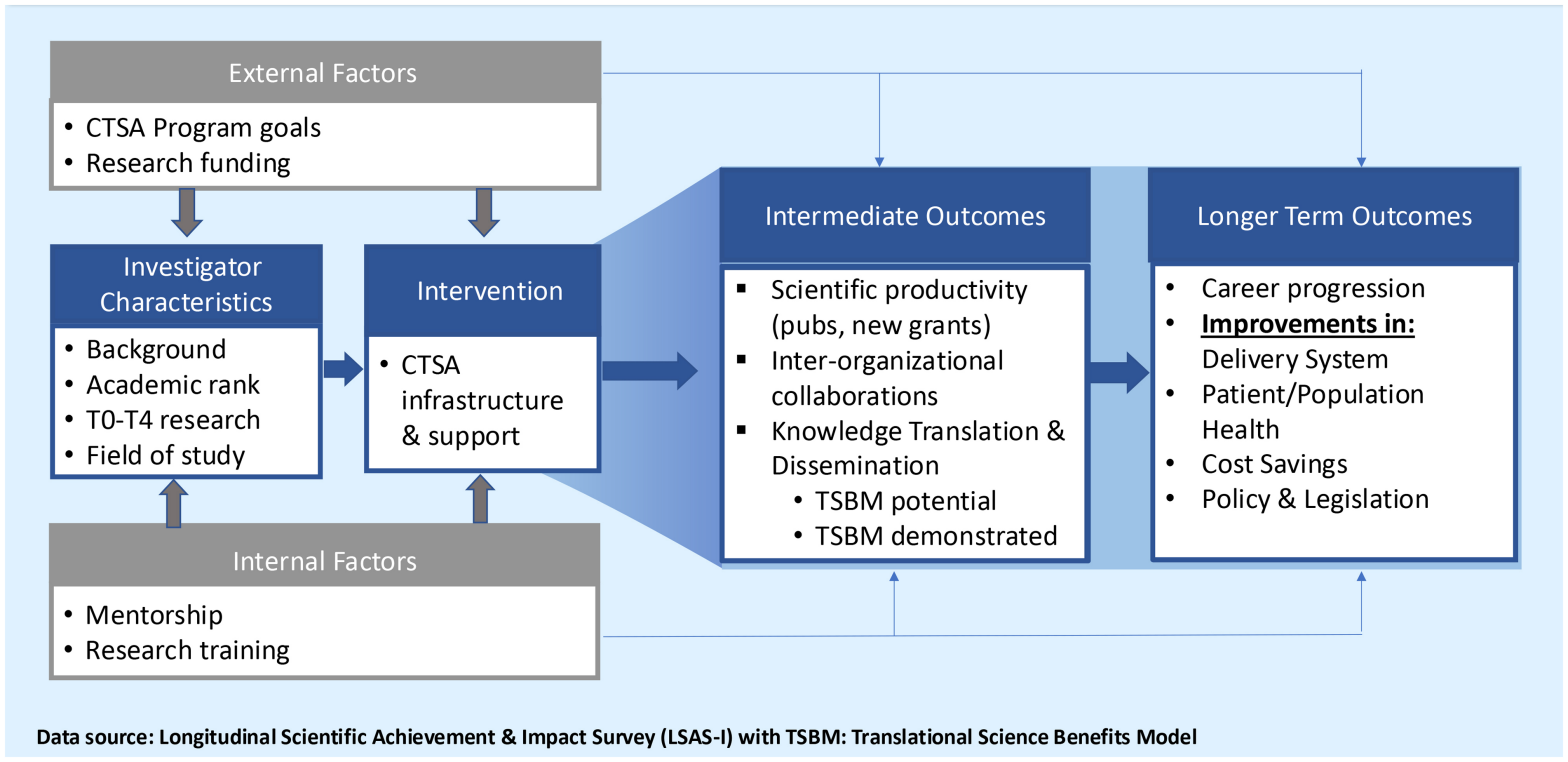
CTSI framework for evaluating Scientific Achievement Translational Science Impact (SATSI).

CTSI-Evaluation continuously engages in hub-level evaluation, using the CTSI Framework to evaluate Scientific Achievement Translational Science Impact (SATSI). The Figure 2 framework shows how the CTSA infrastructure and support lead to intermediate and long-term outcomes. Intermediate outcomes are investigator-centric, focusing on scientific achievement (publications and grants), and research centric focusing on interorganizational collaborations, “potential” and “demonstrated” impact of new grant awards, and dissemination and implementation. Longer-term outcomes include career progression of investigators and improvements in the delivery system, patient and population health, costs, and policy and legislation. The new knowledge generated by the Framework is used to make recommendations for continuously improving the hub infrastructure and operations to innovate and accelerate clinical translational science (Figure 2).

Major data sources used for the evaluation include: (1) quantitative LSAS-I: longitudinal scientific achievement and impact survey, (2) evaluation master database (EMD), and (3) longitudinal qualitative interviews conducted with principal investigators (PIs) who reported high-impact hub-county collaborations supported by the CTSI. The LSAS-I sampling frame of investigators who received CTSI support was formed when the institution was first launched in 2011–2012 with *n* = 261 investigators, now reaching over *n* = 2,400 in 2024. The EMD is a longitudinal data repository which contains information on all CTSI support and services provided to each investigator (e.g., consulting hours, NIH grant writing workshops, pilot awards, bioinformatics data provisioning, Clinical Translational Research centers, CTRC).

Over the years, LSAS-I has been continuously reviewed and updated to keep pace with CTSA Program priorities and innovations. In the Supplementary material for this study, the TSBM checklist provides a description of each of the 30 indicators. Before incorporating TSBM, we collected open-ended qualitative data on impact that was challenging to code and analyze. LSAS-I now generates systematic data on investigator characteristics, types of research (e.g., NCATS priority areas, T0-T4 bench-to-bedside-to population health translational research), interorganizational collaborations, publications, the number and type of new grants and industry support attributable to CTSI, and the reported TSBM knowledge translation impact of the new grants (Figure 2 SATSI).

Collection of both quantitative and qualitative data were used to assess TSBM knowledge translation impact. The 4 domains of the TSBM include 30 quantifiable indicators of knowledge translation impact and the option of indicating either a demonstrated and/or potential benefit per indicator. However, qualitative interviewing was required to understand the impacts on improving health and healthcare in a manner more in-depth than what the TSBM indicators can provide alone. Indicators give us frequencies across a wide range of projects and an opportunity to systematically identify specific types of impact (e.g., computer software development/AI), while the longitudinal interviews give us a rich in-depth understanding summarized in the three impact stories which are presented in the results section.

**Figure 3:**
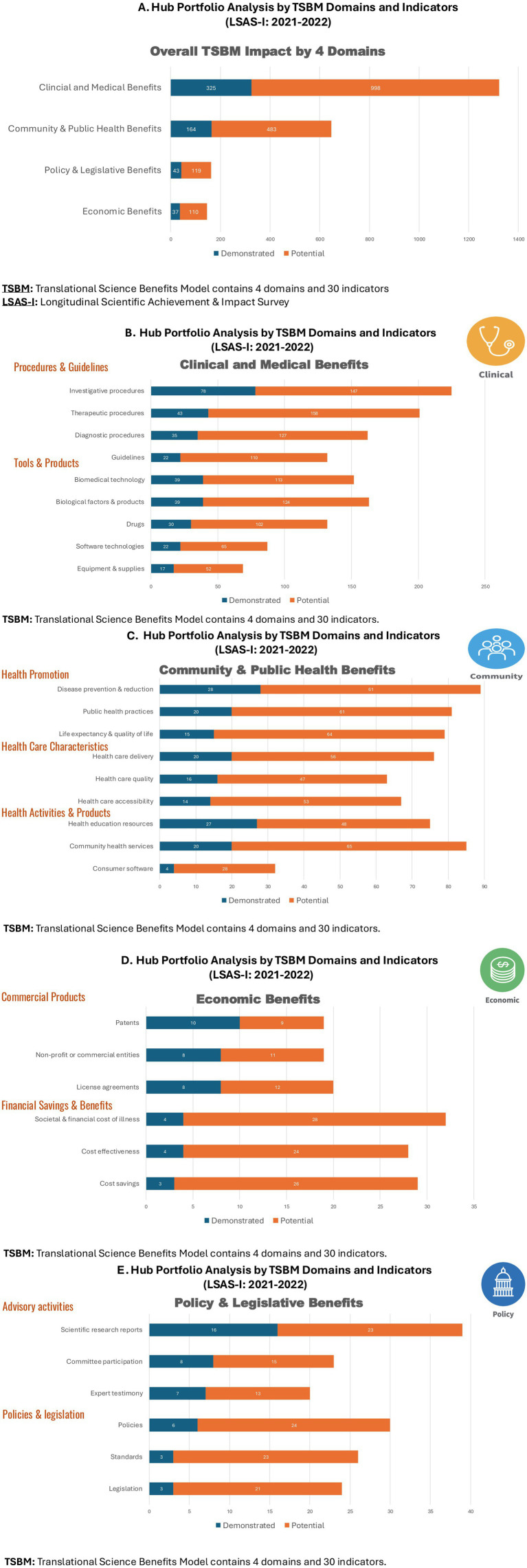
**(A–E)** Hub portfolio analysis by TSBM domains and indicators (LSAS-I: 2021–2022).

To understand hub-level impact, we analyzed TSBM demonstrated and potential knowledge translation using a novel hub portfolio analysis. All new grants attributed to CTSI support were reported by investigators in the LSAS-I: 2021 and 2022, with 2021 being the first year for collecting the TSBM impact data at the UCLA hub. The hub portfolio analysis reports results by the 4 TSBM domains, subdomains, and the 30 indicators (Figure 3).

Subsequently, we focused on the subset of hub-county interorganizational collaborations using a systematic 5-point plan drawing upon both quantitative and qualitative data analyses. Inclusion criteria determined by our hub evaluation office counts interorganizational collaborations which: (i) were supported by the CTSI, and (ii) yielded a new grant from that support and included an LAC collaboration. While Table 1 presents the national context for hub-county interorganizational collaborations (*n*=61 hubs), Table 2 (see “Results” section) presents the 18 new grants and the associated interorganizational collaborations reported in the LSAS-I with some minor augmentation reported in PI interviews.

**Table 1:** National context: CTSA hub-county interorganizational collaborations (2024 internet search).

Number of hub-county research collaborations	0	1	2	3 or more	Total
Frequency	1 (1.6%)	7 (11.5%)	23 (37.7%)	29 (47.5%)	61 (100%)

**Table 2:** CTSI hub-Los Angeles County (LAC) interorganizational collaborations (*n* = 18 projects with 21 interorganizational collaborations).

Grants/projects (*n* = 18)	Safety net hospitals^1^	LAUSD^2^	LAC DHS^3^	LAC DPH^4^	LAC DMH^5^	Other^6^	Total
Dementia diagnoses in a safety-net population			1				1
Mitigating toxic stress response in patients with ACE-related health conditions (obesity management in a community clinic setting)			1				1
A mixed methods evaluation of assisted outpatient treatment in LAC					1		1
Midcareer award in patient-oriented community-academic partnered aging research (K24)						1	1
The impact of youth incarceration on health in adulthood						2	2
Healthy tomorrow’s partnership for children program						1	1
Impact of covid-19 testing and mitigation on return-to-school in the second largest US school district		1		1			2
Leveraging school environments to shape social networks and improve adolescent health: a randomized trial of a social network intervention		1					1
LCIRN: life course intervention research network, scholar’s pilot program (qualitative interviews)		1					1
RAD-X underserved populations safe return to school diagnostic testing		1					1
Cedars Sinai Bairey-Merz lab collaborating with the LAC Department of Public Health	1						1
Effectiveness and implementation of the care ecosystem during COVID-19	1		1				2
Trauma focused traumatic stress evaluation/management for adolescents with posttraumatic stress	1						1
UCLA undiagnosed diseases network clinical site	1						1
Overcoming sleep apnea with mild vibration	1						1
UCLA clinical site undiagnosed disease network: admin supplement 1	1						1
UCLA clinical site undiagnosed disease network: admin supplement 2	1						1
UCLA-UCI center for eliminating cardio-metabolic disparities in multi-ethnic populations	1						1
Total collaboration types (*n* = 21)	8	4	3	1	1	4	21

^1^LA County Safety Net Hospitals (i.e., Olive View-UCLA Medical Center). ^2^LAUSD, LA Unified School District. ^3^LAC DHS, Los Angeles County Department of Health Services. ^4^LAC DPH, Los Angeles County Department of Public Health. ^5^LAC DMH, Los Angeles County Department of Mental Health. ^6^Other (i.e., Parks and Recreation, LA City Department of Aging, Department of Probation, Sheriff’s Department).

The systematic process involved: (i) selecting all the PIs and new grants supported by the CTSI that included an LAC interorganizational collaboration, (ii) summarizing characteristics of the investigators and their research projects; (iii) counting the total number of demonstrated impacts for each research project, (iv) interviewing PIs with the highest number of demonstrated impacts, and finally, (v) using the results to create an Impact Library for research training to build capacity for accelerating translational science.

## 3 Results

### 3.1 National context: CTSA hub-county interorganizational collaborations

Table 1 summarizes findings from the CTSA Program (*n* = 61 hubs) internet search, showing almost 50% (47.5%) of hubs had three or more research collaborations with county agencies, 38% had two collaborations, 11.5% had one collaboration, and only 1 hub reported no collaborations on their website in 2024. By utilizing the ranking methodology documented in the methods section, we ensured that our findings were grounded in a systematic, transparent, and reproducible evaluation process, which lent credibility to establish that the UCLA CTSI hub was among the leading institutions in interorganizational collaborations with county governmental agencies.

### 3.2 Hub portfolio analysis showing demonstrated and potential impact

This study combines 2 years of LSAS-I data (2021, 2022). In the 2-year reporting period, 507 new grants were reported by CTSI investigators. Of these 390 new grants (77% of the 507) reported demonstrated and/or potential impact.

Figure 3A shows the graphic hub portfolio of new grants reporting demonstrated and potential impact by the 4 TSBM domains: (1) clinical and medical, (2) community and public health, (3) economic, and (4) policy and legislative. Underlying the 4 domains are the 30 indicators. In the Supplementary material for this study, the TSBM checklist provides a description of each of the 30 indicators. For each new grant, investigators reported whether each indicator had a potential or demonstrated impact. A grant can have multiple TSBM domains and indicators, in other words the categories are not mutually exclusive.

Figure 3A shows the overall TSBM Impact by the 4 Domains. By far, clinical and medical benefits were the most often reported with 325 demonstrated and 998 potential impacts. These data were collected early in the research incubation period, so it is not surprising that almost 1,000 impacts were “potential” over the grant implementation period. In addition to clinical and medical, the other domains are presented in descending order of knowledge translation impact: community and public health (164 demonstrated, 483 potential), policy and legislative (43 demonstrated, 119 potential), and economic (37 demonstrated, 110 potential).

Figure 3B shows the 2 sub-domains (Procedures and Guidelines, Tools and Products) and the 9 indicators categorized as clinical and medical in the TSBM. Under Procedures and Guidelines, investigative procedures (78 demonstrated, 147 potential) and therapeutic procedures (43 demonstrated, 158 potential) were reported most frequently by the investigators. Under Tools and Products, biomedical technology (39 demonstrated, 113 potential) and biological factors and products (39 demonstrated, 124 potential) were reported most frequently by the investigators. Similarly Figure 3C (Community and Public Health), Figure 3D (Economic) and Figure 3E (Policy and Legislative) report the demonstrated and potential impact for each domain, subdomain, and underlying indicators. A similar pattern emerges throughout the Figure 3 graphics with smaller numbers of demonstrated and greater numbers of potential impact reported. Again, this is due to the reporting of TSBM impact early in the incubation period.

### 3.3 Hub-LAC interorganizational collaborations and impact stories

In the methods section, we described the systematic process for identifying high impact hub-LAC collaborations. We started by selecting all the new grants and associated investigators who were supported by the CTSI that also included an LAC collaboration in the 2-year period (2021–2022). Eighteen new research projects with 21 hub-county collaborations met our criteria for inclusion in the study. Table 2 summarizes the type of county collaboration with the highest percentages reported for county safety net hospitals (38%), LAC unified school district (19%), and smaller percentages of other county health departments, and varying mix of other.

In terms of characteristics of projects and PIs (data not shown), 67% (*n* = 12) were based at UCLA and 17% (*n* = 3) were based at The Lundquist Research Institute. Regarding academic rank, 50% of the PIs were senior, 28% assistant, and 22% associate-level investigators. Not surprisingly, more than 50% of the PIs reported their T0-T4 research areas as T3 (delivery system) and T4 (patient and population health).

Among the 18 hub-county collaborations, 3 PIs and projects were selected for more intensive longitudinal interviews to document the impact stories. These 3 impact stories were selected based on the highest number of demonstrated impacts reported by the PIs within the TSBM’s four domains and 30 indicators within the domains.

The first collaborative project was conducted by Dr. Naser Ahmadi, a physician with specialties in psychiatry and biobehavioral sciences. He reported the highest demonstrated impact in the TSBM Clinical and Medical domain. The collaboration was formed between the CTSI hub and Olive View Medical Center, a Los Angeles County safety net hospital serving a low socioeconomic population with high percentages of Medi-Cal insurance (California’s State Medicaid Program). His research has led to new software technology and Artificial Intelligence (AI) which allows more universal screening for high-risk individuals in emergency room care. Dr. Ahmadi created a screening tool to identify risk factors, protective factors, and outcome measures of adolescent suicide. A new app using AI physiology markers (audiovisual measures) was created to identify high-risk individuals, possible treatment plans, and prediction of how they would respond to each treatment plan. The impact on health and healthcare is a rapid intervention (within 2 days), that can be implemented in any community emergency room, to quickly identify and prevent suicide in high-risk adolescents.

The second collaborative project was conducted by Dr. Elizabeth Barnert, a pediatrician who has emerged as a national expert in identifying the needs and pathways for reentry of incarcerated youth. She reported the highest demonstrated impact in the TSBM Legislative and Policy domain. Based at the CTSI hub, Dr. Barnert formed collaborations with LA County Departments of Mental Health, Health Services, Probation, and the Sheriff’s Department. Her legislative interest is to improve the healthcare delivery system by increasing access and continuity of care and successful reentry. This was achieved by creating partnerships between community providers and the juvenile legal system so that youth in conflict with the law and/or survivors of child sex trafficking can have better medical and mental health services. In the longer-term Dr. Barnert’s research emphasizes the creation of developmental pathways associated with better physical and mental health as they grow into adulthood.

**Figure 4:**
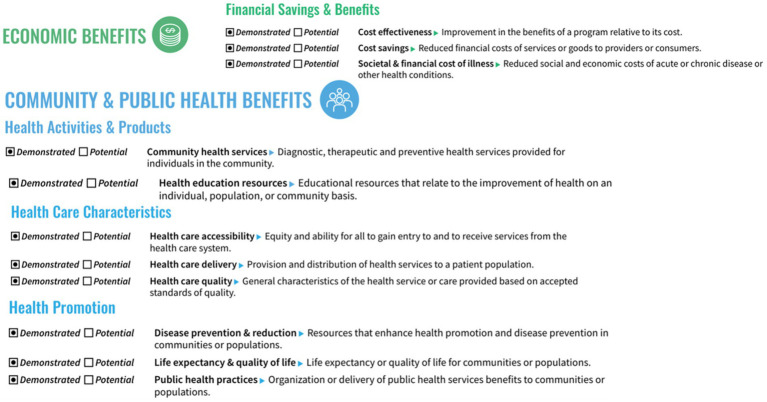
TSBM economic and community and public health impact.

The third collaborative project was spearheaded by Dr. Catherine Sarkisian, a geriatrician and NIH-funded research scientist. Dr. Sarkisian’s “K24 Midcareer Investigator Award in Patient-Oriented Aging Research,” evolved into the UCLA Healthcare Value Analytics and Solutions (UVAS), a new consultation service and a portfolio of novel research projects. Dr. Sarkisian reported the highest impact in the TSBM Economic indicators by implementing effective interventions to reduce costs and improve health care practice. As illustrated, Figure 4 shows demonstrated economic benefit of this research on three TSBM indicators: (i) cost effectiveness, (ii) cost savings, and (iii) society and financial cost of illness. UVAS illuminates the substantial economic benefits of various quality improvement initiatives. Utilizing plan-do-study-act cycles, a quality improvement nurse reviewed medical records and educated staff with data on overuse of preoperative medical visits, chest x-rays, laboratory tests, and electrocardiograms. The intervention was found to have projected savings of $67,000 over 3 years for LAC-DHS facilities.

## 4 Discussion

The study reports baseline TSBM knowledge transfer data for the initial 2 years of our TSBM data collection (LSAS-I: 2021–2022) at the UCLA hub. In addition to looking at overall TSBM impact, we took a deeper dive into hub-county interorganizational collaborations to learn more about their effects on health care delivery systems and patient and population health improvement. This study examines TSBM impact in hub-county collaborations; no other studies were found in the peer-reviewed literature that looked at impact in a systematic way.

The TSBM baseline data were collected early in the incubation period. Of the 507 new grants, 77% of investigators reported TSBM impact. Nevertheless, TSBM knowledge translation impact is still a relatively new and unknown concept at our hub. Greater effort will be required to hardwire and widely implement TSBM into research training and career development to accelerate translational science innovations and successful knowledge transfer, ultimately leading to improvements in health and healthcare. Additionally, some hubs, e.g., Washington University St. Louis CTSA, are using TSBM as a foundation for dissemination and implementation (D&I) planning and execution (11). We expect this trend to increase in the CTSA Program as TSBM continues to be tested as a foundation for these efforts.

Across the national CTSA program, almost 50% of hubs were found to report three or more interorganizational collaborations with county health agencies. Prior to this research we had no systematic knowledge about the national landscape for hub-county collaborations or the impact of these collaborations. Further we did not understand how our hub ranked in this national context. The data from the 61 hub website searches clearly underestimates the number of interorganizational collaborations. In fact, in the 2021–2022 reporting period, our hub had 18 hub-county collaborations and most of these were ongoing. These collaborations are not reported on our website but rather captured in our LSAS-I annual surveys. Additionally, Figure 1 shows the substantial opportunities for hub-county health innovation and intervention projects and the potential for multisite clinical and translational science research collaborations with Los Angeles County.

We also note that 50% of the PIs of the UCLA hub collaborations were senior investigators, and the majority of PIs (> 50%) indicated their translational research was conducted in the T3-T4 translation space (clinical implementation and public health). The increasing number and type of interorganizational collaborations suggest an emerging trend for hubs across the nation to more seriously pursue research and development (R&D) to improve county delivery systems and patient and population health, particularly among uninsured or underinsured patients who utilize the county safety net system.

### 4.1 Generalizability of study methods and findings

Generalizability is concerned with the wider applicability of the findings across geographic locations, settings, populations, disease conditions, public health and health promotion interventions. Measuring impact is increasingly critical for any research enterprise—large, small, or even a smaller portfolio of sponsored research projects. Impact measures are a pivotal tool to demonstrate that continued investment is worthwhile and serves as a constructive indicator of ROI.

The results of our study are generalizable in at least two ways: (i) hub portfolio analysis to examine Scientific Achievement Translational Science Impact (SATSI), and (ii) hub-county interorganizational collaborations to improve health and healthcare. This study is innovative in that it applies a standardized methodology, TSBM checklist as a data collection mechanism in our annual LSAS-I survey, to produce comparable data across research settings and projects, a key aspect of generalizability. In terms of best practices in translational science impact measurement, the TSBM alone does not fully capture all essential dimensions. It is equally important to consider the research organization in terms of structures, operations, and innovations whether large-scale, small-scale, or a portfolio of sponsored research projects—that influence outcomes and impact. Hence, our evaluation designs must also measure structural and process innovations and opportunities for continuous quality improvement.

Additionally, we need to test research training activities to increase awareness of the growing importance of impact and measurement using the TSBM model that can also be used as a foundation for building dissemination and implementation (D&I) strategies (11–14). Finally, our study provides a valuable framework for assessing interorganizational collaborations within the national CTSA program. The methods of this study and findings are applicable to the 60 + hubs and across other research infrastructures.

Beyond the UCLA hub, our findings have broader implications for improving national reporting. NCATS leadership may consider embedding the 30 TSBM indicators into the hub annual reports (RPPR) to systematically track knowledge translation impact. These data reports could be aggregated to: (i) assess strengths and gaps in translational science outcomes nationwide, and (ii) build a database of contextual variables to examine factors influencing knowledge translation impact, such as the number and type of interorganizational collaborations (e.g., multi-CTSA partnerships, hub-county health initiatives, and hub partner institutions).

### 4.2 Future studies opportunities and challenges

Moreover, our findings suggest limited TSBM activity within the economic, and policy and legislative domains. Future research and development might focus on these gaps and test interventions and approaches to increase the impact of translational science in these domains. Addressing these gaps would not only strengthen the CTSA Program but also advance real-world impact of research by improving how scientific discoveries are translated into medical practice, the delivery system, community-based interventions, and public health policies.

While we relied on an internet search to quantify county collaborations, future research could be strengthened by conducting a systematic survey to gather direct responses from each CTSA hub. For example, in addition to constructing contextual variables, targeted items could be added to an existing CTSA Program Evaluators Survey to enhance our understanding of the national context of the CTSA Program, such as the role of interorganizational collaborations in influencing patient access, research outcomes, and broader systemic impact.

Finally, although hub portfolio analysis offers promising methodology, our findings do not represent the complete demonstrated impact of the hub. Results show SATSI reported over only 2-years (2021–2022). Currently, we collect TSBM data from investigators early in the incubation period. Thus, baseline data reported here underestimates the true knowledge translation impact of our hub.

## Data Availability

The raw data supporting the conclusions of this article will be made available by the authors, without undue reservation.

## Footnotes

^1^Hunt J. Personal Communication Indiana Clinical and Translational Science Institute (CTSI) (2025) (unpublished).
