# Supplementary material for: Clinical and translational science award hub portfolio analysis and interorganizational collaborations with Los Angeles County to improve population health and health care delivery (ID 1565096)
Source: Front Public Health. 2025 Jun 19;13:1565096. doi: 10.3389/fpubh.2025.1565096 (PMC12222129; doi:10.3389/fpubh.2025.1565096)
Supplement: Supplementary file 1 [file Data_Sheet_1.PDF]

TSBM Benefits (Indicators) Checklist

CLINICAL COMMUNITY ECONOMIC POLICY

Use this checklist to track the CLINICAL, COMMUNITY, ECONOMIC, & POLICY benefits of your work.

Many research projects will have clinical and translational research benefits—or indicators—in multiple domains, as noted in the Translational Science Benefits Model (TSBM).

The indicators are specific, observable, and are each at least potentially measurable. Demonstrated benefits are those that have been observed and are verifiable; potential benefits are those logically expected with moderate to high confidence.

For each benefit demonstrated by your project, please choose EITHER demonstrated or potential, and not both.

CLINICAL & MEDICAL BENEFITS

Procedures & Guidelines

- ☐ *Demonstrated* ☐ *Potential* — **Diagnostic procedures** ▸ Methods and techniques performed to diagnose disease, disorders, or conditions.
- ☐ *Demonstrated* ☐ *Potential* — **Investigative procedures** ▸ Research methods used in pre-clinical, clinical, and other scientific studies.
- ☐ *Demonstrated* ☐ *Potential* — **Guidelines** ▸ Formal recommendations or principles to assist with patient care for specific clinical circumstances.
- ☐ *Demonstrated* ☐ *Potential* — **Therapeutic procedures** ▸ Methods and techniques that pertain to interventions, treatment, or prevention of diseases, disorders or conditions.

Tools & Products

- ☐ *Demonstrated* ☐ *Potential* — **Biological factors & products** ▸ Biological substances used to indicate, diagnose, prevent, or treat diseases or medical conditions.
- ☐ *Demonstrated* ☐ *Potential* — **Biomedical technology** ▸ Technology applications for solving medical problems.
- ☐ *Demonstrated* ☐ *Potential* — **Drugs** ▸ Pharmaceutical products for human or veterinary use.
- ☐ *Demonstrated* ☐ *Potential* — **Equipment & supplies** ▸ Apparatus, instruments, and materials for diagnostic, surgical, therapeutic, and scientific procedures.
- ☐ *Demonstrated* ☐ *Potential* — **Software technologies** ▸ Computer programs or software installed on mobile or other electronic devices.

COMMUNITY & PUBLIC HEALTH BENEFITS

Health Activities & Products

- ☐ *Demonstrated* ☐ *Potential* — **Community health services** ▸ Diagnostic, therapeutic and preventive health services provided for individuals in the community.
- ☐ *Demonstrated* ☐ *Potential* — **Consumer software** ▸ Digital and mobile technologies used by or for consumers to improve health care delivery and outcomes.
- ☐ *Demonstrated* ☐ *Potential* — **Health education resources** ▸ Educational resources that relate to the improvement of health on an individual, population, or community basis.

Health Care Characteristics

- ☐ *Demonstrated* ☐ *Potential* — **Health care accessibility** ▸ Equity and ability for all to gain entry to and to receive services from the health care system.
- ☐ *Demonstrated* ☐ *Potential* — **Health care delivery** ▸ Provision and distribution of health services to a patient population.
- ☐ *Demonstrated* ☐ *Potential* — **Health care quality** ▸ General characteristics of the health service or care provided based on accepted standards of quality.

TSBM Benefits (Indicators) Checklist

Health Promotion

☐ *Demonstrated* ☐ *Potential* **Disease prevention & reduction ▶** Resources that enhance health promotion and disease prevention in communities or populations.

☐ *Demonstrated* ☐ *Potential* **Life expectancy & quality of life ▶** Life expectancy or quality of life for communities or populations.

☐ *Demonstrated* ☐ *Potential* **Public health practices ▶** Organization or delivery of public health services benefits to communities or populations.

ECONOMIC BENEFITS

Commercial Products

☐ *Demonstrated* ☐ *Potential* **License agreements ▶** Governmental permits based on intellectual property. Researchers develop new technologies to be used for diagnostic or therapeutic applications with possible commercial use. Examples of applications are vaccines, drug deliveries, medical devices, imaging, software, algorithms, blood and tissue products, cellular and gene therapy, among others. A license allows for exploration of applications for potential human benefit.

☐ *Demonstrated* ☐ *Potential* **Non-profit or commercial entities ▶** Creation of businesses or non-profit agencies.

☐ *Demonstrated* ☐ *Potential* **Patents ▶** Government authority or licenses based on intellectual property.

Financial Savings & Benefits

☐ *Demonstrated* ☐ *Potential* **Cost effectiveness ▶** Improvement in the benefits of a program relative to its cost.

☐ *Demonstrated* ☐ *Potential* **Cost savings ▶** Reduced financial costs of services or goods to providers or consumers.

☐ *Demonstrated* ☐ *Potential* **Societal & financial cost of illness ▶** Reduced social and economic costs of acute or chronic disease or other health conditions.

POLICY & LEGISLATIVE BENEFITS

Advisory Activities

☐ *Demonstrated* ☐ *Potential* **Committee participation ▶** Participation in advisory, standards, or other governmental or nongovernmental committees.

☐ *Demonstrated* ☐ *Potential* **Expert testimony ▶** Presentation of data or results to governmental, judicial, or other regulatory bodies.

☐ *Demonstrated* ☐ *Potential* **Scientific research reports ▶** Research findings or summaries provided to inform policy or regulatory activity.

Policies & Legislation

☐ *Demonstrated* ☐ *Potential* **Legislation ▶** Bills, laws, statutes, and ordinances passed through formal legislative bodies such as congress, state legislatures, and county and city councils.

☐ *Demonstrated* ☐ *Potential* **Policies ▶** Procedural rules formally adopted and mandated by governmental agencies or private or non-profit organizations. These include what are commonly referred to as big “P” policies (governmental) and little “p” policies (non-governmental organizational).

☐ *Demonstrated* ☐ *Potential* **Standards ▶** Formal designations of levels of quality defined by industry, occupational groups, or governmental bodies.
